# Blood transfusion in total knee arthroplasty and total hip arthroplasty: A nationwide study of complications, costs and predictive modelling

**DOI:** 10.1002/jeo2.70317

**Published:** 2025-07-13

**Authors:** David Maman, Maneesh Nandakumar, Michael T. Hirschmann, Hadas Ofir, Madlene Haddad, Butrus Samir, Yaniv Steinfeld, Yaron Berkovich

**Affiliations:** ^1^ Technion Israel Institute of Technology Haifa Israel; ^2^ Department of Orthopedics Carmel Medical Center Haifa Israel; ^3^ School of Medicine and Public Health University of Newcastle Callaghan New South Wales Australia; ^4^ Logan Hospital Brisbane Queensland Australia; ^5^ Department of Orthopaedic Surgery and Traumatology Kantonsspital Baselland (Bruderholz, Liestal, Laufen) Bruderholz Switzerland

**Keywords:** arthroplasty, blood transfusion, healthcare costs, machine learning, outpatient surgery, predictive modelling, surgical outcomes

## Abstract

**Purpose:**

Blood transfusion during total knee and hip arthroplasty is associated with increased postoperative complications, prolonged hospital stays and greater healthcare costs. As outpatient arthroplasty expands, identifying patients at high transfusion risk is essential. This study analyses over 4 million arthroplasty procedures from the Nationwide Inpatient Sample (NIS) to assess the clinical and economic impact of transfusion and develop a machine learning‐based risk prediction tool. We hypothesised that transfused patients would experience higher complication rates, longer hospital stays and increased hospitalisation costs.

**Methods:**

We conducted a retrospective cohort study using NIS data (2016–2019) including primary total knee arthroplasty and total hip arthroplasty cases. Propensity score matching (PSM) was used to balance clinical and demographic variables. Outcomes included length of stay (LOS), total charges, complications and mortality. Logistic regression, random forest and deep neural networks (DNNs) were trained to predict transfusion using preoperative data. Validation methods included hold‐out testing, class weighting and dropout.

**Results:**

After matching, transfusion was linked to increased surgical site infection (TKA RR = 17.0; THA RR = 13.5), sepsis (TKA RR = 13.4; THA RR = 5.0) and pulmonary embolism (TKA RR = 6.0; THA RR = 3.5). Transfused patients had longer LOS (TKA: 4.2 vs. 2.7 days; THA: 4.0 vs. 2.9 days) and higher charges (TKA: $79,996 vs. $59,600; THA: $89,283 vs. $77,239). The DNN achieved the best predictive performance (area under the curve: 0.8644–0.8783). Top preoperative predictors of transfusion included chronic anaemia, chronic kidney disease, female gender and osteoporosis.

**Conclusions:**

Blood transfusion significantly worsens clinical outcomes and increases costs in arthroplasty. Our machine learning tool, while not clinically implemented yet, shows promise in identifying high‐risk patients and supporting preoperative planning.

**Level of Evidence:**

Level III.

**Abbreviations**
AI: artificial intelligence
AUC: area under the curve
BCEWithLogitsLoss: binary cross entropy with logits loss
CI: confidence interval
DNN: deep neural network
EHR: electronic health record
HCUP: healthcare cost and utilisation project
ICD‐10: International Classification of Diseases, 10th Revision
IRB: institutional review board
LOS: length of stay
NIS: nationwide inpatient sample
PSM: propensity score matching
RR: risk ratio
SPSS: Statistical Package for the Social Sciences
THA: total hip arthroplasty
TKA: total knee arthroplasty

## INTRODUCTION

Blood transfusion is a critical yet challenging aspect of perioperative management, particularly in total knee arthroplasty (TKA) and total hip arthroplasty (THA) [1, 2, 3, 6, 7, 24, 25]. While transfusions can be lifesaving in certain situations, they also pose significant risks, including infection, immune reactions and thromboembolic complications [3, 6, 7, 10, 11, 24]. Additionally, the necessity of blood transfusion imposes logistical constraints, as it requires access to a blood bank and trained personnel [3, 4, 5]. This requirement inherently limits the feasibility of performing arthroplasties in outpatient surgical centres or smaller clinics that lack transfusion capabilities.

A key challenge in optimising surgical outcomes and healthcare resource allocation is the ability to preoperatively identify patients at high risk for transfusion. Early identification of these patients allows for targeted interventions, such as preoperative haemoglobin optimisation and intraoperative blood conservation strategies, which may reduce the need for transfusion and mitigate associated risks [1, 6, 7, 8, 16, 17]. Conversely, patients with a low risk of transfusion could be considered for surgery in settings without transfusion capabilities, facilitating same‐day discharge and reducing overall costs [2, 9, 21, 22, 26].

In this study, we utilised a large national inpatient dataset to develop and validate predictive models for blood transfusion in TKA and THA patients. Using machine learning techniques, we trained models on preoperative variables—including demographics and comorbidities—to generate transfusion risk predictions. We evaluated multiple models, including logistic regression, random forest and deep neural networks (DNNs), assessing their accuracy and clinical applicability. Additionally, we developed a user‐friendly software tool to enable real‐time estimation of transfusion risk based on individual patient characteristics. This study uses machine learning techniques to generate individualised transfusion risk predictions based on preoperative data. While our findings are preliminary, such tools may support surgical triage decisions and optimise perioperative care planning.

The goal of this study is to enhance perioperative blood management by providing an objective, data‐driven approach for identifying patients at risk for transfusion. By leveraging machine learning for individualised risk prediction, our findings may contribute to improved surgical planning, reduced transfusion‐related complications, and although not assessed directly in this study, such tools may eventually aid in selecting appropriate candidates for ambulatory arthroplasty settings, pending further validation.

## MATERIALS AND METHODS

### Dataset acquisition

This study utilised data from the nationwide inpatient sample (NIS), the largest publicly available all‐payer inpatient care database in the United States. Patients undergoing TKA and THA were identified using ICD‐10 procedure codes. The dataset covered the period from 1 January 2016, to 31 December 2019. The NIS, part of the Healthcare Cost and Utilisation Project (HCUP), captures approximately 20% of all inpatient hospitalisations, providing a representative sample of over seven million unweighted hospitalisations annually. This analysis included more than 2.5 million TKA procedures and more than 1.6 million THA procedures.

### Patient identification and exclusions

Patients undergoing primary TKA and THA were identified based on ICD‐10 procedure codes. To ensure a homogeneous study population, patients with nonelective admissions, those under 18 years of age, and those undergoing revision arthroplasty were excluded. Patients requiring blood transfusion during hospitalisation were identified using ICD‐10 codes associated with transfusion procedures. The final dataset was stratified into patients with and without blood transfusion, forming the basis for subsequent analyses.

### Statistical analyses and propensity score matching (PSM)

All statistical analyses were conducted using SPSS 26 and MATLAB 2024. Baseline demographic and clinical characteristics were compared using independent *t*‐tests for continuous variables and chi‐square tests for categorical variables. A *p*‐value < 0.05 was considered statistically significant.

To minimise confounding and ensure a balanced comparison, two separate PSM analyses were conducted—one for TKA and one for THA—using MATLAB. A one‐to‐one nearest‐neighbour matching algorithm was applied, based on a logistic regression model incorporating demographic and clinical variables. Variables included in the matching process were age, gender, insurance status (Medicare, Medicaid, private insurance, self‐pay or other), hospital size, hospital region, race and average income in the hospital area, along with comorbidities. The PSM process resulted in two well‐matched cohorts for each procedure, ensuring comparability between patients with and without blood transfusion.

### Feature selection for machine learning models

We started with a large administrative dataset of patients undergoing either hip or knee replacement surgery. To ensure that our predictive model relied solely on preoperative information, we removed any hospital‐related variables such as location and bed size, as well as post‐surgical details including discharge status and length of stay (LOS). The final feature set comprised the patient's age, sex, and comorbid conditions extracted from ICD‐10 codes. These comorbidities were converted into binary features (1 = present, 0 = absent) and grouped by the first three characters of the ICD‐10 code to reduce dimensionality. Additionally, low‐prevalence comorbidities with minimal association with transfusion risk were filtered out.

### Machine learning models for blood transfusion prediction

To develop predictive models for blood transfusion risk, three machine learning algorithms were trained and evaluated: logistic regression, random forest and DNN. Logistic regression modelled the log‐odds of the outcome, while random forest utilised an ensemble of decision trees aggregated via majority voting. The DNN consisted of four layers, including three hidden layers and one output layer, incorporating batch normalisation and dropout after each hidden layer to prevent overfitting. In the DNN, class weighting was applied in the loss function using BCEWithLogitsLoss, which combines a Sigmoid activation and binary cross‐entropy loss into a single function.

Hyperparameter tuning for the DNN was performed using Optuna, optimising key parameters that influence the model's learning capacity and generalisation. These included the number of units in each hidden layer (u1, u2, u3), which determine the model's ability to capture complex patterns; the dropout rates (d1, d2, d3), which help prevent overfitting by randomly deactivating neurons during training; and the learning rate (lr), which controls how quickly the model updates its parameters during optimisation. The final hyperparameters were determined separately for hip and knee procedures. For hip replacements, the model used u1 = 64, u2 = 64, u3 = 48, d1 = 0.3, d2 = 0.5, d3 = 0.3 and lr = 0.001. For knee replacements, the model used u1 = 128, u2 = 64, u3 = 64, d1 = 0.2, d2 = 0.5, d3 = 0.5 and lr = 0.001.

To validate the models and reduce overfitting, dropout layers and class weighting were used in the neural network, and performance was evaluated using hold‐out validation. Sensitivity analyses were performed using unmatched regression models and by varying the PSM caliper. A key limitation of the NIS database is the lack of intraoperative variables such as estimated blood loss, operative time and type of anaesthesia, which were therefore not included in the predictive models.

### Development of the blood transfusion prediction software

A user‐friendly blood transfusion prediction software was developed, integrating the trained machine learning models. The interface allows users to select the type of surgery (TKA or THA), choose the predictive model (logistic regression, random forest or DNN), and input patient‐specific characteristics such as age, gender and ICD‐10 comorbidities. Once the information is entered, the model generates a probability estimate for blood transfusion risk. Additionally, users can clear patient data for new predictions. This tool provides an accessible way to integrate predictive modelling into clinical decision‐making, allowing for individualised transfusion risk assessment.

### Ethical considerations

This study was exempt from institutional review board (IRB) approval, as it utilised de‐identified patient data from the NIS database, which does not contain protected health information. Informed consent was not required.

## RESULTS

### Demographic, payer characteristics and comorbidity comparison between TKA patients with and without blood transfusion

As shown in Table 1, patients who required blood transfusion accounted for 1.2% of all TKA procedures. These patients were significantly older, with an average age of 70.0 years compared to 66.7 years in those who did not receive a transfusion (*p* < 0.001). The proportion of female patients was also higher in the transfusion group (74.0% vs. 61.4%, *p* < 0.001).

**Table 1:** Demographic, payer characteristics and comorbidity comparison in total knee arthroplasty patients with and without blood transfusion.

Parameters in total knee arthroplasty	No blood transfusion	Blood transfusion	Significance
Total surgeries	2,484,329 (98.8%)	29,770 (1.2%)	‐
Average age (years)	66.7	70.0	*p* < 0.001
Female (%)	61.4	74.0	*p* < 0.001
Primary expected payer—Medicare (%)	56.9	68.8	*p* < 0.001
Primary expected payer—Medicaid (%)	4.2	4.8	
Primary expected payer—private including Health Maintenance Organization (%)	35.2	23.6	
Primary expected payer—self‐pay (%)	0.5	0.5	
Primary expected payer—no charge (%)	0	0.1	
Primary expected payer—other (%)	3.1	2.2	
Hypertension (%)	59.6	56.9	*p* < 0.001
Dyslipidemia (%)	46.6	49.4	*p* < 0.001
Chronic anaemia (%)	5.6	15.6	*p* < 0.001
Obstructive sleep apnoea (%)	13.2	10.3	*p* < 0.001
Alcohol abuse (%)	0.9	1.2	*p* < 0.001
Osteoporosis (%)	3.9	7.1	*p* < 0.001
Chronic kidney disease (%)	6.8	16.3	*p* < 0.001
Congestive heart failure (%)	1.2	3	*p* < 0.001
Chronic lung disease (%)	5.9	8.8	*p* < 0.001
Diabetes mellitus (%)	21.6	27.8	*p* < 0.001
Liver disease (%)	1.2	1.8	*p* < 0.001
Fibromyalgia (%)	2.7	3	*p* = 0.002
Disorders of thyroid (%)	17.8	22.9	*p* < 0.001
Obesity (%)	31.1	26.1	*p* < 0.001

Regarding payer characteristics, Medicare was the primary expected payer for a significantly larger proportion of patients receiving transfusions (68.8% vs. 56.9%, *p* < 0.001), while private insurance was less common in this group (23.6% vs. 35.2%). Medicaid coverage was slightly higher in the transfusion group (4.8% vs. 4.2%), whereas self‐pay and uninsured rates showed minimal differences.

All assessed comorbidities, except obesity, were more prevalent in patients who required blood transfusion (26.1% vs. 31.1%, *p* < 0.001).

### Demographic, payer characteristics and comorbidity comparison between THA patients with and without blood transfusion

As shown in Table 2, patients who required blood transfusion accounted for 2.9% of all THA procedures. These patients were significantly older, with an average age of 68.1 years compared to 65.4 years in those who did not receive a transfusion (*p* < 0.001). The proportion of female patients was also notably higher in the transfusion group (71.1% vs. 54.9%, *p* < 0.001).

**Table 2:** Demographic, payer characteristics and comorbidity comparison in total hip arthroplasty patients with and without blood transfusion.

Parameters in total hip arthroplasty	No blood transfusion	Blood transfusion	Significance
Total surgeries	1,587,835 (97.1%)	47,780 (2.9%)	‐
Average age (years)	65.4	68.1	*p* < 0.001
Female (%)	54.9	71.1	*p* < 0.001
Primary expected payer—Medicare (%)	54.4	65.5	*p* < 0.001
Primary expected payer—Medicaid (%)	5	6.4	
Primary expected payer—private including Health Maintenance Organization (%)	37.5	25.2	
Primary expected payer—self‐pay (%)	0.7	0.7	
Primary expected payer—no charge (%)	0.1	0.1	
Primary expected payer—other (%)	2.3	2.1	
Hypertension (%)	52.2	51.4	*p* = 0.002
Dyslipidemia (%)	42.3	42.7	*p* = 0.08
Chronic anaemia (%)	5.6	12.1	*p* < 0.001
Obstructive sleep apnoea (%)	10.1	9.1	*p* < 0.001
Alcohol abuse (%)	1.5	2.1	*p* < 0.001
Osteoporosis (%)	4.5	9.2	*p* < 0.001
Chronic kidney disease (%)	6.3	13.3	*p* < 0.001
Congestive heart failure (%)	1.2	2.7	*p* < 0.001
Chronic lung disease (%)	6.5	11.4	*p* < 0.001
Diabetes mellitus (%)	14.8	19	*p* < 0.001
Liver disease (%)	1.2	2.1	*p* < 0.001
Fibromyalgia (%)	2	3.1	*p* < 0.001
Disorders of thyroid (%)	15.5	19.8	*p* < 0.001
Obesity (%)	23.3	20	*p* < 0.001

Regarding payer characteristics, Medicare was the primary expected payer for a significantly larger proportion of patients in the transfusion group (65.5% vs. 54.4%, *p* < 0.001), while private insurance was less common (25.2% vs. 37.5%). Medicaid coverage was slightly higher among those who received a transfusion (6.4% vs. 5%), whereas self‐pay and uninsured rates showed minimal differences.

Similar to TKA, all assessed comorbidities, except obesity (20.0% vs. 23.3%, *p* < 0.001), were more prevalent in patients who required blood transfusion. Dyslipidemia showed a slightly higher prevalence in the transfusion group (42.7% vs. 42.3%), but this difference was not statistically significant (*p* = 0.08).

### Propensity score‐matched analysis of comorbidities in TKA and THA patients with and without blood transfusion

To minimise baseline differences and reduce potential confounding, a propensity score‐matched analysis was conducted for patients undergoing TKA and THA with and without blood transfusion. This methodology ensured that the two groups were matched based on key demographic and clinical characteristics, creating comparable groups for analysis.

#### TKA

Following PSM, the final cohort included 29,768 (50.0%) patients without blood transfusion and 29,770 (50.0%) patients who received a blood transfusion. There were no statistically significant differences between the groups.These results confirm that the matching process effectively balanced baseline characteristics, reducing potential confounding in the analysis of clinical outcomes.

#### THA

For the THA cohort, the final propensity score‐matched groups consisted of 29,768 (50.0%) patients without blood transfusion and 29,770 (50.0%) patients who received a blood transfusion. The matched groups showed no significant differences in age, gender, payer characteristics and comorbidities.

The results from Tables 3 and 4 demonstrate that the propensity score‐matching methodology effectively created balanced cohorts, allowing for a more reliable and unbiased comparison of clinical outcomes in TKA and THA patients with and without blood transfusion.

**Table 3:** Propensity score‐matched comparison of demographics, payer characteristics and comorbidities in total knee arthroplasty patients with and without blood transfusion.

Parameters in total knee arthroplasty	No blood transfusion	Blood transfusion	Significance
Total surgeries	29,768 (50.0%)	29,770 (50.0%)	‐
Average age (years)	70.0	70.0	*p* = 0.94
Female (%)	74.0	74.0	*p* = 0.91
Primary expected payer—Medicare (%)	69	68.8	*p* = 0.07
Primary expected payer—Medicaid (%)	4.6	4.8	
Primary expected payer—private including Health Maintenance Organization (%)	23.8	23.6	
Primary expected payer—self‐pay (%)	0.2	0.5	
Primary expected payer—no charge (%)	0.1	0.1	
Primary expected payer—other (%)	2.3	2.2	
Hypertension (%)	56.7	56.9	*p* = 0.68
Dyslipidemia (%)	49.4	49.4	*p* = 0.97
Chronic anaemia (%)	15.5	15.6	*p* = 0.70
Obstructive sleep apnoea (%)	10	10.3	*p* = 0.24
Alcohol abuse (%)	0.9	1.2	*p* = 0.05
Osteoporosis (%)	7	7.1	*p* = 0.63
Chronic kidney disease (%)	16.5	16.3	*p* = 0.44
Congestive heart failure (%)	2.9	3	*p* = 0.40
Chronic lung disease (%)	8.2	8.8	*p* = 0.09
Diabetes mellitus (%)	27.9	27.8	*p* = 0.75
Liver disease (%)	1.4	1.8	*p* = 0.16
Fibromyalgia (%)	2.9	3	*p* = 0.47
Disorders of thyroid (%)	23.1	22.9	*p* = 0.06
Obesity (%)	26.2	26.1	*p* = 0.85

**Table 4:** Propensity score‐matched comparison of demographics, payer characteristics and comorbidities in total hip arthroplasty patients with and without blood transfusion.

Parameters in total hip arthroplasty	No blood transfusion	Blood transfusion	Significance
Total surgeries	29,768 (50.0%)	29,770 (50.0%)	‐
Average age (years)	68.2	68.1	*p* = 0.35
Female (%)	71.3	71.1	*p* = 0.71
Primary expected payer—Medicare (%)	64.8	65.5	*p* = 0.09
Primary expected payer—Medicaid (%)	6.4	6.4	
Primary expected payer—private including Health Maintenance Organization (%)	26.3	25.2	
Primary expected payer—self‐pay (%)	0.7	0.7	
Primary expected payer—no charge (%)	0.1	0.1	
Primary expected payer—other (%)	1.7	2.1	
Hypertension (%)	51.6	51.4	*p* = 0.67
Dyslipidemia (%)	42.8	42.7	*p* = 0.92
Chronic anaemia (%)	12	12.1	*p* = 0.84
Obstructive sleep apnoea (%)	9	9.1	*p* = 0.43
Alcohol abuse (%)	2	2.1	*p* = 0.73
Osteoporosis (%)	9.2	9.2	*p* = 0.82
Chronic kidney disease (%)	13.3	13.3	*p* = 1
Congestive heart failure (%)	2.6	2.7	*p* = 0.06
Chronic lung disease (%)	11.1	11.4	*p* = 0.06
Diabetes mellitus (%)	19.3	19	*p* = 0.34
Liver disease (%)	2.3	2.1	*p* = 0.12
Fibromyalgia (%)	3.1	3.1	*p* = 0.80
Disorders of thyroid (%)	20	19.8	*p* = 0.11
Obesity (%)	19.9	20	*p* = 0.80

### Propensity score‐matched analysis of outcomes in TKA and THA patients with and without blood transfusion

Following PSM, the impact of blood transfusion on key clinical and financial outcomes was evaluated in patients undergoing TKA and THA, as shown in Table 5.

**Table 5:** Propensity score‐matched comparison of clinical and financial outcomes in total knee arthroplasty (TKA) and total hip arthroplasty (THA) patients with and without blood transfusion.

Surgery	Parameter	No blood transfusion	Blood transfusion	Significance
TKA	Length of stay mean in days	2.7 (Std. deviation 1.2)	4.2 (Std. deviation 2.9)	*p* < 0.001
	Total charges mean in $	59,600 (Std. deviation 33,671)	79,996 (Std. deviation 52,384)	*p* < 0.001
	Mortality in %	0%	0.3%	*p* < 0.001
THA	Length of stay mean in days	2.9 (Std. deviation 2.6)	4.0 (Std. deviation 3.8)	*p* < 0.001
	Total charges mean in $	77,239 (Std. deviation 57,787)	89 283 (Std. deviation 61 063)	*p* < 0.001
	Mortality in %	0.2%	0.2%	*p* = 0.49

#### TKA outcomes

Patients who required blood transfusion had a significantly longer hospital stay compared to those who did not (4.2 vs. 2.7 days, *p* < 0.001). Additionally, the mean total hospital charges were notably higher in the transfusion group, increasing from $59,600 (±$33,671) to $79,996 (±$52,384), reflecting a $20,396 difference (*p* < 0.001). Mortality was also significantly higher in the transfusion group.

#### THA outcomes

Similarly, in THA patients, blood transfusion was associated with a longer hospital stay (4.0 vs. 2.9 days, *p* < 0.001). The mean total hospital charges were also significantly increased, rising from $77,239 (±$57,787) in the nontransfusion group to $89,283 (±$61,063) in the transfusion group, representing a $12,044 increase (*p* < 0.001). However, unlike in TKA, mortality rates were not significantly different between the two groups.

In TKA, a small but significant increase in mortality was observed among transfused patients. However, this pattern was not evident in THA. This discrepancy may be related to lower transfusion rates, patient selection, or power differences between cohorts.

### Risk ratios of perioperative complications in TKA and THA patients following blood transfusion

**Figure 1:**
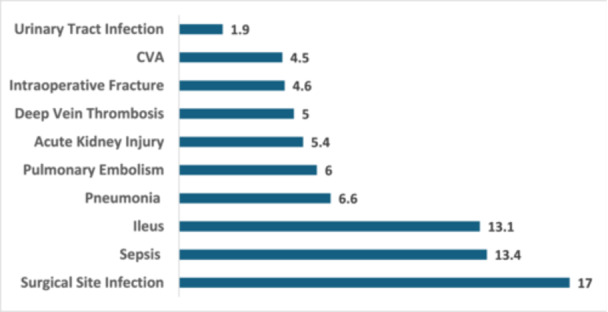
Elevated risk ratios for perioperative complications in total knee arthroplasty patients following blood transfusion after propensity score matching.

Figure 1 illustrates the increased risk of perioperative complications in TKA patients who require blood transfusion, based on propensity score‐matched analysis. Surgical site infection had the highest risk ratio (relative risk [RR] = 17, 95% confidence interval [CI]: 6.9–42), indicating a 17‐fold increased risk in the transfusion group. Sepsis (RR = 13.4, 95% CI: 7.9–22.7) and ileus (RR = 13.1, 95% CI: 7.7–22.1) also showed a significantly elevated risk. Other notable complications included pneumonia (RR = 6.6, 95% CI: 4.9–8.8) and pulmonary embolism (RR = 6.0, 95% CI: 4.5–7.9). All findings were statistically significant with *p* < 0.001.

**Figure 2:**
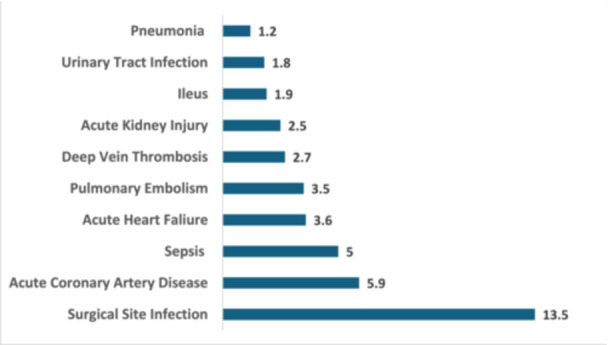
Elevated risk ratios for postoperative complications in total hip arthroplasty patients following blood transfusion after propensity score matching.

Figure 2 presents the relative risk of complications in THA patients who require blood transfusion. Surgical site infection (RR = 13.5, 95% CI: 7.1–25.7) remained the most strongly associated complication, similar to TKA. Cardiovascular complications such as acute coronary artery disease (RR = 5.9, 95% CI: 4.4–8) and acute heart failure (RR = 3.6, 95% CI: 2.8–4.8) were significantly elevated in transfused THA patients. Sepsis (RR = 5.0, 95% CI: 3.8–6.6) and pulmonary embolism (RR = 3.5, 95% CI: 2.6–4.7) showed a strong association with transfusion. Deep vein thrombosis (RR = 2.7, 95% CI: 2.3–3.3) and acute kidney injury (RR = 2.5, 95% CI: 2.3–2.6) were notably higher in transfused THA patients. The risk of pneumonia (RR = 1.2, 95% CI: 1.0–1.4) was elevated but lower than in TKA patients. Again, all findings were statistically significant with *p* < 0.001.

Although relative risk estimates were statistically significant, some values—such as RR = 17 for surgical site infection—are exceptionally high and may reflect residual confounding, miscoding, or limitations in administrative coding systems. These should be interpreted cautiously.

### Prediction models for blood transfusion in TKA and THA

To identify patients at risk for blood transfusion following TKA and THA, we developed machine learning models trained on patient demographic, clinical and procedural data. Table 6 presents the performance metrics of three predictive models: Logistic regression, random forest and DNN.

**Table 6:** Blood transfusion prediction models performance metrics.

Model	AUC (THA)	Acc (THA)	Recall (THA)	AUC (TKA)	Acc (TKA)	Recall (TKA)
Logistic regression	0.8642	78.80%	0.79, 0.81	0.8810	80.13%	0.80, 0.85
Random forest	0.8560	79.46%	0.79, 0.79	0.8708	81.10%	0.81, 0.82
Deep neural network	0.8644	81.90%	0.82, 0.7865	0.8783	82.81%	0.8282, 0.8209

Abbreviations: AUC, area under the curve; THA, total hip arthroplasty; TKA, total knee arthroplasty.

Among the models, DNN demonstrated the highest overall performance, with an area under the curve (AUC) of 0.8644 for THA and 0.8783 for TKA. It also achieved the highest accuracy, 81.90% for THA and 82.81% for TKA, outperforming both logistic regression and random forest. Logistic regression, while slightly lower in accuracy, showed strong AUC values (0.8642 for THA and 0.8810 for TKA) and recall rates comparable to the other models. Random forest performed well, though it had slightly lower AUC values compared to the other methods.

The trained model will be available for download alongside the article, allowing for further validation and potential clinical implementation. To better interpret these values, AUC ratings can be classified into different performance categories. An AUC between 0.50 and 0.60 is considered poor, while values from 0.60 to 0.70 indicate fair performance. AUC scores ranging from 0.70 to 0.80 are classified as good, whereas those between 0.80 and 0.90 are rated as very good. Models achieving an AUC of 0.90 to 1.00 are considered excellent. In this study, all models demonstrated AUC values within the very good range (0.85–0.88), indicating strong predictive performance.

### Blood transfusion prediction software for hip and knee arthroplasty

**Figure 3:**
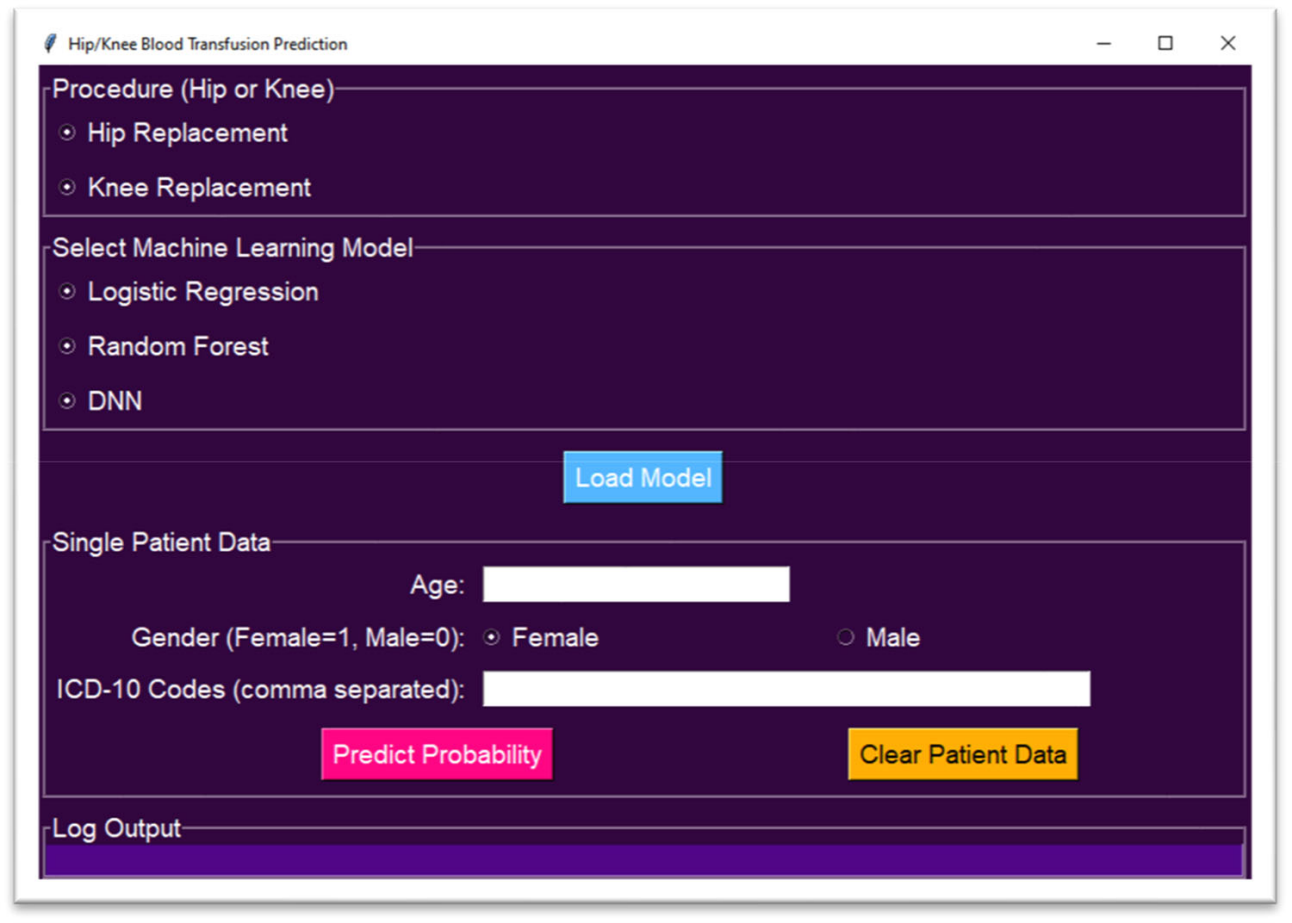
Blood transfusion prediction software for hip and knee arthroplasty.

Figure 3 displays the user interface of the blood transfusion prediction software, which allows clinicians to estimate the probability of transfusion for patients undergoing TKA or THA. Users can select the procedure type (hip or knee arthroplasty) and choose from three different machine learning models.

The software enables input of patient‐specific data, including age, gender and ICD‐10 codes for comorbid conditions. Once this information is entered, users can load the selected model and click the ‘predict probability’ button to obtain an estimated likelihood of blood transfusion for the given patient. Additionally, there is an option to clear patient data for new predictions.

This tool provides an accessible way to integrate predictive modelling into clinical decision‐making.

## DISCUSSION

### Key findings

Our results indicate that blood transfusion is associated with a measurable increase in postoperative complications and healthcare expenditures. Although these findings are consistent with existing literature, our study adds a large‐scale analysis with modern predictive modelling.

### Clinical and economic impact of blood transfusion in arthroplasty

A blood A blood transfusion is one of the most vital interventions in orthopaedic surgery but also a high‐risk procedure that increases morbidity, length of hospital stay and financial burden. Studies have shown that transfusions increase complication rates, including surgical site infections, sepsis and cardiovascular events, with complication rates rising from 13.2% in nontransfused patients to 34.7% in transfused patients [12, 18]. Additionally, the length of hospitalisation increases significantly—by up to 1.5 days for TKA and 1.1 days for THA—and hospital costs rise to $20,396 for TKA and $12,044 for THA [13].

Thus, individual risk stratification to mitigate transfusion‐related complications with improved perioperative care is of immediate necessity. Risk factors for transfusion include preoperative anaemia, low BMI, female gender and prolonged operative time [9, 23]. From a health economics perspective, the ability to predict the risk of transfusion preoperatively offers an opportunity for reducing costs and improving resource efficiency.

As global demand for arthroplasty continues to grow, data‐driven strategies to optimise blood management may enable personalised, targeted interventions—such as preoperative correction of anaemia—to minimise unnecessary transfusions and associated complications. Machine learning models offer an efficient, scalable real‐time solution to these challenges by providing clinicians with objective, patient‐specific risk assessments.

### Artificial intelligence (AI) in digital medicine: advancing personalised risk prediction

The DNN model demonstrated strong performance (AUC 0.8644–0.8783), comparable to traditional methods [14, 15, 16, 23, 24]. However, differences between DNN and logistic regression were modest, and we caution against overstating the advantages of AI without further clinical validation.

The software tool we developed is a research prototype, not a certified clinical product. Its use in real‐world settings will require usability testing, regulatory approvals and prospective evaluation.

To ensure explainability, we employed Shapley Additive Explanations (SHAP) values to interpret model outputs, allowing clinicians to understand the relative contribution of variables such as age, sex and comorbid conditions to transfusion risk. This improves transparency and aligns with digital medicine's emphasis on interpretable AI.

This tool, while not yet deployed in clinical practice, represents a potential step toward data‐supported triage planning and optimisation of blood resource use, as previously explored in digital health frameworks [8, 9, 10]. The prediction model has not yet been shown to reduce transfusion rates or change outcomes. For orthopaedic surgeons, this tool could assist in triaging patients preoperatively—particularly in deciding whether same‐day discharge is feasible, or whether preoperative interventions such as anaemia management should be prioritised. While not yet used clinically, the model offers a step toward practical, data‐driven support for optimising perioperative care in everyday practice.

### Expanding the boundaries of arthroplasty: The case for outpatient surgery

One of the most significant implications of our predictive model is its potential to support outpatient and ambulatory arthroplasty [15, 25, 28]. The requirement for transfusion‐capable facilities is a primary constraint in transitioning joint replacement procedures to nonhospital settings [2, 16, 26]. Our AI‐driven risk assessment tool enables accurate identification of low‐risk patients, providing an evidence‐based framework for selecting candidates suitable for same‐day discharge or ambulatory surgery [8, 14, 27, 28, 29].

The expansion of outpatient arthroplasty carries profound clinical and economic benefits, including reduced healthcare costs, lower infection risks, and increased patient convenience. By leveraging AI for perioperative risk stratification, we offer a pathway for hospitals and surgical centres to safely implement outpatient arthroplasty, optimising both clinical outcomes and healthcare resource utilisation.

### Strengths and limitations

A major strength of this study is its foundation on a nationally representative dataset, ensuring broad generalisability. The integration of AI into predictive modelling further enhances the reliability and clinical utility of our risk assessment tool. Additionally, our software platform represents a direct clinical translation of machine learning research, bridging the gap between theoretical modelling and real‐world surgical applications.

However, several limitations should be acknowledged. First, our study relies on administrative data, which may be subject to coding inaccuracies [19, 20, 21, 22] and lacks detailed intraoperative variables such as blood loss and fluid management. Additionally, all comorbidities were extracted from ICD‐10 codes and treated as binary variables (present/absent), without severity quantification, which limits the granularity of risk assessment. Second, although our models demonstrate strong predictive accuracy, external validation in prospective cohorts is necessary to confirm their real‐world effectiveness. Third, the implementation of our AI‐based tool into clinical practice requires further evaluation to assess its impact on surgical planning, patient safety and cost‐effectiveness. Notably, essential clinical factors that influence transfusion risk—such as preoperative haemoglobin levels, use of tranexamic acid, antiplatelet therapy, tourniquet use and surgical approach—are not available in the NIS dataset, which may impact the comprehensiveness of our model.

Additionally, bias in AI‐driven decision‐making remains a concern. To address this, we conducted subgroup analyses to assess model performance across demographic groups, ensuring equitable predictions across age, sex and socioeconomic categories. Future work should focus on further refining bias mitigation strategies to enhance fairness in AI‐driven perioperative care.

### Future directions

To further advance AI‐powered perioperative risk prediction, we plan to initiate real‐world validation of our model in a clinical setting. This will involve retrospective validation using institutional data from a high‐volume orthopaedic centre, followed by prospective testing within an academic hospital [9, 10, 14, 27]. Integration into the electronic health record (EHR) system is also planned to enable seamless, automated risk assessment at the point of care. This step is crucial for assessing the practical utility of our AI model in surgical decision‐making, optimising blood management strategies and refining deployment for widespread clinical use.

To further advance AI‐powered perioperative risk prediction, future research should focus on prospective validation across diverse healthcare environments, including high‐volume academic centres and ambulatory surgical facilities. Integration with EHR could enable seamless, automated risk assessment, enhancing clinical efficiency. Additionally, AI‐guided interventions for transfusion risk reduction, such as targeted anaemia management protocols, represent a promising avenue for improving patient outcomes.

Another potential research direction is the expansion of predictive modelling beyond blood transfusion, incorporating broader perioperative risk assessments such as venous thromboembolism, surgical site infections and acute kidney injury. AI‐driven risk stratification could redefine surgical decision‐making, paving the way for more precise, individualised perioperative care.

## CONCLUSION

This study quantifies the increased burden of blood transfusion in arthroplasty and explores the potential use of AI in risk stratification. Our predictive model demonstrates technical feasibility but requires clinical validation. As the use of AI in perioperative care evolves, rigorous evaluation will be necessary to confirm its impact on surgical decision‐making and patient outcomes.

## AUTHOR CONTRIBUTIONS

David Maman performed the statistical analysis, data interpretation and contributed to the manuscript writing. Maneesh Nandakumar contributed to the majority of the manuscript writing and editing. Michael Tobias Hirschmann, Yaron Berkovich and Yaniv Steinfeld assisted in writing the manuscript and provided senior mentorship and clinical interpretation throughout the project. Hadas Ofir, Madlene Haddad and Butrus Samir contributed to the AI model development, data processing and integration of the AI‐driven tool into the study. Yaron Berkovich supervised the entire project and served as the principal investigator for all stages of the research. All authors reviewed and approved the final version of the manuscript.

## CONFLICT OF INTEREST STATEMENT

The authors declare no conflicts of interest.

## ETHICS STATEMENT

This study was performed in compliance with the Declaration of Helsinki. It was exempt from IRB review, and the requirement for consent was waived. The study was conducted under an exempt status granted by the Institutional Review Board (IRB). Due to the use of a de‐identified dataset from the National Inpatient Sample (NIS), the requirement for informed consent was waived. The research adhered to the ethical principles outlined in the Declaration of Helsinki. Consent was not required for this study, as it utilised a de‐identified dataset, with no direct human participation involved that would necessitate consent.

## Supporting information

Supporting Information – AI Blood.zip This supplementary ZIP file includes the blood transfusion prediction software used in this study. The file contains: GUI_BloodTransfusion.py – the main Python script for launching the graphical user interface (GUI). Readme.txt – a user guide explaining how to run the software and interpret results. pkl_files/– contains three subfolders (dnn, logistic_regression, and random_forest), each with the respective trained machine learning models used for prediction.

## Data Availability

The dataset analysed during the current study is available for purchase from the Healthcare Cost and Utilisation Project (HCUP) National Inpatient Sample (NIS), sponsored by the Agency for Healthcare Research and Quality (AHRQ), at www.hcup‐us.ahrq.gov.
